# Single-cell RNA sequencing combined with whole exome sequencing reveals the landscape of the immune pathogenic response to chronic mucocutaneous candidiasis with STAT1 GOF mutation

**DOI:** 10.3389/fimmu.2022.988766

**Published:** 2022-09-15

**Authors:** Xiaodi Lu, Keming Zhang, Weiwei Jiang, Hang Li, Yue Huang, Mingwei Du, Jian Wan, Yanyun Cao, Lin Du, Xiaogang Liu, Weihua Pan

**Affiliations:** ^1^ Shanghai Key Laboratory of Molecular Medicine Mycology, Naval Medical University, Shanghai, China; ^2^ Department of Dermatology, 72nd Group Army Hospital of People’s Liberation Army (PLA), Huzhou, China; ^3^ Department of Dermatology, Pudong New Area People’s Hospital, Shanghai, China; ^4^ Shanghai Key Laboratory of Pathogenic Fungi Medical Testing, Pudong New Area People’s Hospital, Shanghai, China

**Keywords:** chronic mucocutaneous candidiasis (CMC), signal transducer and activator of transcription 1 (STAT1), heterozygous mutation, single-cell RNA sequencing (scRNAseq), immune mechanism

## Abstract

Chronic mucocutaneous candidiasis (CMC) is characterized by recurrent or persistent infections with *Candida* of the skin, nails, and mucous membranes (*e.g.*, mouth, esophagus, and vagina). Compared with that of other infectious diseases, the immune pathogenic mechanism of CMC is still poorly understood. We identified a signal transducer and activator of transcription 1 gain-of-function (c.Y289C) mutation in a CMC patient. Single-cell transcriptional profiling on peripheral blood mononuclear cells from this patient revealed decreases in immature B cells and monocytes. Further analysis revealed several differentially expressed genes related to immune regulation, including RGS1, TNFAIP3, S100A8/A9, and CTSS. In our review of the literature on signal transducer and activator of transcription 1 gain-of-function (c.Y289C) mutations, we identified seven cases in total. The median age of onset for CMC (n=4, data lacking for three cases) was 10.5 years (range: birth to 11 years), with an average onset age of 8 years. There were no reports linking tumors to the c.Y289C mutation, and the incidence of pre-existing clinical disease in patients with the c.Y289C mutation was similar to previous data.

## Introduction

Chronic mucocutaneous candidiasis (CMC) is characterized by recurrent or persistent infections of the skin, nails, and mucous membranes (*e.g.*, mouth, esophagus, and vagina) caused by the fungal species, *Candida (* 1). Most CMC cases are sporadic and secondary to other underlying diseases, such as HIV infection, leading to T cell immunodeficiency and diabetes, which are treated with immunosuppressive or steroid therapy. CMC rarely has a familial/hereditary origin and is mainly caused by congenital immunodeficiency, usually associated with autosomal dominant or recessive mutations in a single gene. The virulence genes linked with CMC include signal transducer and activator of transcription 1 (*STAT1*), signal transducer and activator of transcription 1 (*STAT3*), *IL-17F*, *IL-17RA/RC*, ACT1 (*TRAF3IP2*), Caspase recruitment domain protein 9 (*CARD9*), retinoic acid-related orphan nuclear receptor C (*RORC*), and autoimmune regulator (*AIRE*), among which STAT1 gain-of-function (GOF) mutations account for half of all cases.

In this study, we report a patient with CMC, for whom we identified a STAT1 GOF (c.Y289C) mutation. We applied single-cell RNA Sequencing analysis to unveil changes in peripheral blood mononuclear cells (PBMCs). Because of the rarity of the c.Y289C mutation and the lack of existing data, we hope that this article, and the incorporated literature review, will offer insight into the clinical features of CMC caused by this mutation.

## Materials and methods

### PBMC collection

We collected 5 ml peripheral blood from the CMC patient. By using a standard density gradient centrifugation with Ficoll Paque solution, PBMCs were freshly isolated from EDTA anticoagulated venous blood and cryopreserved for long-term storage and the subsequent generation of a single-cell transcriptome library.

### Whole exome sequencing

In order to eliminate the influence of sequencing errors on the results, it is necessary to perform quality control on the original sequencing data to obtain clean reads. The preprocessing software is fastp (2), and the quality filtering criteria are as follows: (1) remove the linker sequence. (2) Remove reads with N (non-AGCT) bases greater than or equal to 5 (3). Perform a sliding window with four bases as the size of Windows, and cut off the reads of which average base quality value is less than 20 (4). After the above filtering, remove reads whose length is less than 75 bp or whose average base quality value is less than 15.

The genomic DNA in the sample was extracted, and the library was constructed after the electrophoresis test was qualified. Qualified DNA samples were first randomly broken into 150 bp - 220 bp fragments by Covaris, and the Agilent SureSelect Human All Exon V6 Kit was used for library construction and capture. The DNA fragments go through the steps of end repair, adding ployA tails, adding sequencing adapters, purification, magnetic bead capture, PCR amplification, etc., and finally complete the library construction.

BWA (3) was used to align the Clean Reads to the reference genome (reference genome version was GRCh37.p13). After the alignment results were formatted by SAMtools (4), Picard was used to remove redundancy, and Qualimap software was used to compare the results for analysis. SNP and InDel detection was performed using the Haplotypecaller module of the GATK4 (5) software based on the alignment of the samples to the reference genome. Before detecting SNPs and InDels, the base quality is recalibrated using the BaseRecalibrator module of the GATK4 software based on known human SNP and InDel databases to improve the accuracy of variant detection. In order to reduce the error rate of SNP and InDel detection, the standard of QD >= 2.0 was used for filtering, and only the mutation sites that met this condition were retained. QD is the ratio of variance quality (Quality) divided by coverage depth (Depth), which is actually the variance quality value per unit depth. Most of the false positive variances’ QD values ​​are less than 2. SNP and InDel detection results were annotated to Refseq, Thousand Genes, EXAC, esp6500, gnomAD, SIFT, clinvar, PolyPhen, MutationTaster, COSMIC (6), gwasCatalog, OMIM and other databases using Annovar (7) software. We used CNVkit (8) software to detect CNV of the samples and employ Lumpy (9) software to detect SV information on the samples.

### Single-cell RNA-seq

According to the manufacturer’s protocol, Single-cell RNA-seq libraries were prepared using a Chromium Single cell 3′Reagent kit, version 3. Briefly, the beads with cell barcode and cells are wrapped in nanoliter-scale droplets, the droplets containing cells are collected, and then the cells are lysed in the droplets, so that the mRNA in the cells is combined with the cell barcode on the bead to form single cell gel beads in emulsions (GEMs). After generation of GEMs, each cDNA was generated through reverse transcription and added with a cell barcoding sequence and Unique Molecular Identifier (UMI). Then, libraries were constructed and sequenced on the Illumina sequencing platform (Illumina, San Diego, CA).

### Single-cell RNA seq data processing

The Cell Ranger software pipeline (version 3.1.0) provided by 10×Genomics was used to demultiplex cellular barcodes, map reads to the genome and transcriptome using the STAR aligner, and down-sample reads as required to generate normalized aggregate data across samples, producing a matrix of gene counts versus cells. We processed the unique molecular identifier (UMI) count matrix using the R package Seurat (10) (version 3.0). To remove low quality cells and likely multiplet captures, which is a major concern in microdroplet-based experiments, we apply a criteria to filter out cells with UMI/gene numbers out of the limit of mean value +/- 2 fold of standard deviations assuming a Guassian distribution of each cells’ UMI/gene numbers. Following visual inspection of the distribution of cells by the fraction of mitochondrial genes expressed, we further discarded low-quality cells where a certain percentage of counts belonged to mitochondrial genes. Library size normalization was performed in Seurat on the filtered matrix to obtain the normalized count.

Top variable genes across single cells were identified using the method described in Macosko et al. (11). Briefly, the average expression and dispersion were calculated for each gene, genes were subsequently placed into several bins based on expression. Principal component analysis (PCA) was performed to reduce the dimensionality on the log transformed gene-barcode matrices of top variable genes. Cells were clustered based on a graph-based clustering approach, and were visualized in 2-dimension using tSNE. Likelihood ratio test that simultaneously test for changes in mean expression and in the percentage of expressed cells was used to identify significantly differentially expressed genes between clusters. Here, we use the R package SingleR (12), a novel computational method for unbiased cell type recognition of scRNA-seq to infer the cell of origin of each of the single cells independently and identify cell types.

Differentially expressed genes (DEGs) were identified using the Seurat (10)package. P value < 0.05 and |log2foldchange| > 1 (or |log2foldchange| > 0.58) was set as the threshold for significantly differential expression. GO enrichment and KEGG pathway enrichment analysis of DEGs were respectively performed using R based on the hypergeometric distribution.

## Results

### Clinical description of the patient

**Figure 1:**
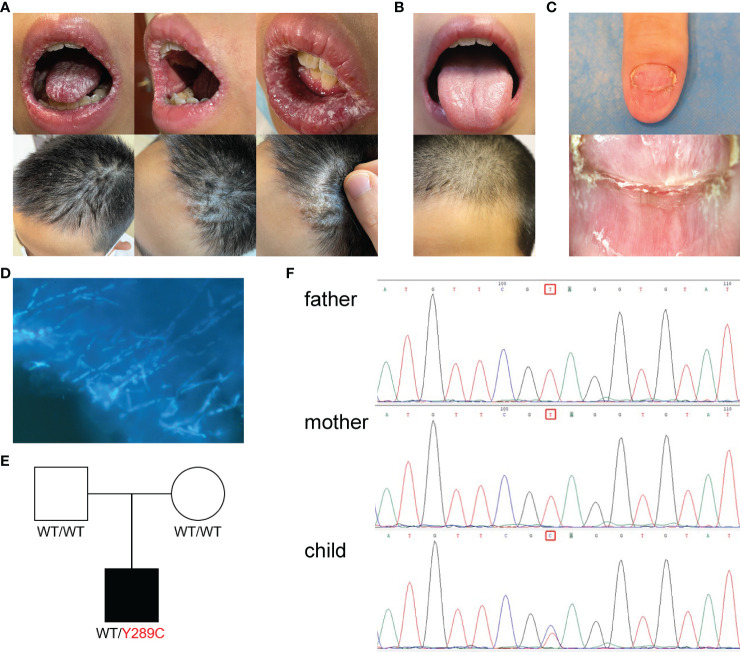
Clinical features, Laboratory tests and genetic tests of a CMC patient. **(A)** Picture of fungal infection in the patient’s oral mucosa and scalp at the first visit. **(B)** Picture of the patient’s oral mucosa and scalp after fluconazole treatment. **(C)** Dermoscope used for the nail. **(D)** Fungal fluorescent staining of the nail: fungal hyphae showed by strong blue fluorescence (×1). **(E)** Pedigree of a patient’s family with segregation of the p.Y289C mutant allele. The patient is denoted by a black symbol. **(F)** Sequencing profiles demonstrating the heterozygous STAT1 c.A866G:p.Tyr289Cys mutation in the patient.

The CMC patient was a 12-year-old male, residing in China, who had been hospitalized twice with a diagnosis of Henoch–Schonlein purpura. He had suffered with mycotic stomatitis since September 2020, and a fungal infection of the thumb on his right hand since January 2021 (**Figure 1A**). After visiting the local children’s hospital, the patient was treated with silver ions spray, and the treatment was interrupted a few weeks later with tiny effective against thrush. In May 2021, the patient visited our hospital, and dermoscopy and fungal fluorescent staining of the nail cleared the *Candida* infection (**Figures 1C, D**). Laboratory tests showed that the levels of serum immunoglobulins (IgG, IgA, IgM, and IgE) and antibodies related to autoimmune diseases were normal, but the level of C4 was slightly high (0.458 g/L, normal range 0.16–0.38 g/L) and positivity to anti-EBV-CAIgG suggested previous Epstein-Barr virus (EBV) infection. Serum CD8+ T cells were increased (35.3%, normal range 18.1%–29.6%) and CD4/CD8 cells were decreased (1.00%, normal range 1.57%–2.93%), which was indicative of abnormal immune and inflammatory responses. Antimicrobial susceptibility tests showed that fungi from three infection sites were sensitive to amphotericin B, fluorocytosine, itraconazole, voriconazole, and fluconazole. The patient was prescribed fluconazole, at 50 mg per day. The white pseudomembrane in the oral cavity disappeared after five days (**Figure 1B**), and the *Candida* infection of the nails recovered over a longer time period. Combined with the patient’s previous history of Henoch–Schonlein purpura and persistent superficial *Candida* infections, the preliminary diagnosis was CMC (1, 13, 14).

### Identification of a homozygous STAT1 GOF mutation

Because the patient’s parents were not consanguineous and there were no similar cases in the family history, we suspected that the patient may have a *de novo* mutation or recessive homozygosity, and the peripheral blood of the patient and his parents was extracted for next-generation sequencing. Whole-exome sequencing revealed a heterozygous mutation in the *STAT1* gene leading to an amino acid substitution of cystine for tyrosine (c.866A > G; p.Y289C). This mutation occurred *de novo* in the patient—neither of his parents carried the variant (**Figure 1E**). Database searches identified previous reports of the same mutation site linked to disease (15, 16). The whole-exome sequencing data were verified by Sanger sequencing, which confirmed the site of the mutation (**Figure 1F**).

STAT1 GOF mutations are usually autosomal dominant, and such mutations usually impair dephosphorylation or hyperphosphorylation of STAT1 in the nucleus, mostly by affecting the coiled-coil domain (CCD) and the DNA-binding domain (DBD). A systematic analysis of a large sample showed that STAT1 GOF mutations located in the DBD may have more severe clinical manifestations (17). Fortunately, the patient’s mutation was located in the CCD and clinical manifestations have been limited to local rather than systemic fungal infection to date.

### Single-cell transcriptomics highlight a shift in immune cell population in CMC

**Figure 2:**
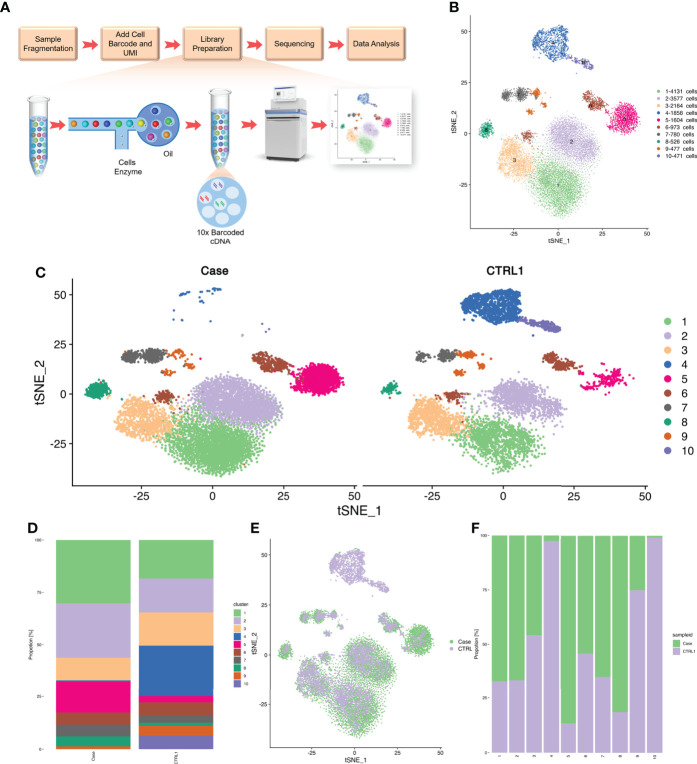
Single cell RNA-seq study design and initial cell grouping. **(A)** Scheme of the scRNA-seq study design. **(B)** The tSNE projection of 9,168 single PBMCs from patient and 7,393 single PBMCs from healthy control, showing the formation of 10 main clusters. Each dot corresponds to one single cell, colored according to cell cluster. **(C)** Global tSNE plots of merged patient and healthy control cells. Patient cells are colorized in the left panel, and healthy control cells are colorized in the right panel. Cell clusters and their respective colors are labeled on the right. **(D)** Distribution of the abundance of each cell in each cell cluster in the patient and healthy control datasets. **(E)** The re-clustered tSNE projection of PBMCs from patient’s samples and healthy control’s samples. The samples are labeled with different colors for each cell. **(F)** Bar plot highlighting the cell abundances across clusters (n=10) for patient and healthy control. UMI, Unique Molecular Identifiers.

**Figure 3:**
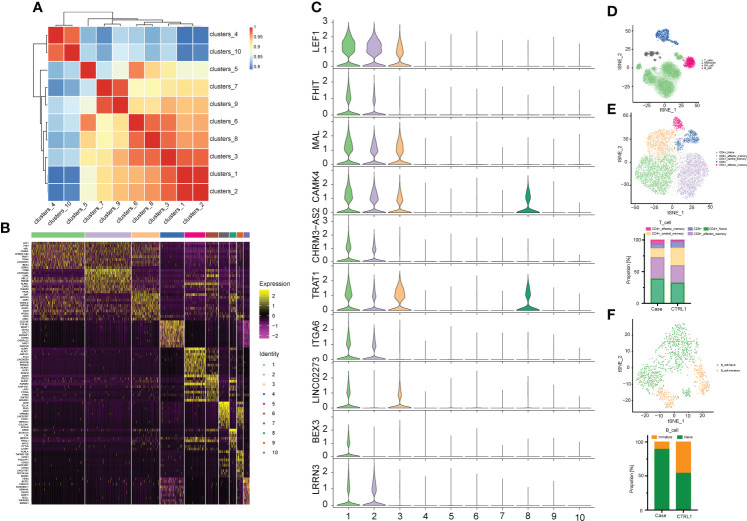
Correlation analysis between cell populations and identification of cell types. **(A)** Heat map representing Pearson correlation between clusters based on overall scRNA-seq dataset. Red and blue colors, indicate positive and negative correlation, respectively. **(B)** Distinct gene signatures (top 10 differentially expressed genes; bimod test) of 10 clusters. **(C)** Violin plot showing expression levels of Top 10 Marker gene in cell cluster 1. **(D)** The tSNE plots of PBMCs from the two datasets. Cell subtypes (T cell, monocyte, NK cell, B cell) are labeled with different colors. **(E)** The above picture show tSNE plots of T cells from the two datasets, and the following picture show distribution of the abundance of each cell in each cell cluster. T cell subtypes are labeled with different colors. **(F)** The above picture show tSNE plots of B cells from two datasets, and the following picture show distribution of the abundance of each cell in each cell cluster. Cell subtypes (naïve B cell and immature B cell) are labeled with different colors.

We isolated PBMCs from the patient’s blood sample obtained at the stage of clinical rescue. With the patient’s sample, 11,430 single cells were captured, compared with 10,191 single cells for the healthy control sample. After quality control by Seurat, transcriptomes of 7,393 and 9,186 single cells from the patient and healthy control, respectively, were acquired. Total cells were classified into 10 different types from cluster 1 to cluster 10 by unbiased clustering (**Figure 2**). Based on the expression of known marker genes, the cell lineages of monocytes, T cells, natural killer (NK) cells, and B cells were identified (**Figures 3A–D**; **Supplementary Figure 1**). T cells were divided into five populations and B cells were divided into two populations, i.e., immature B cells and naive B cells (**Figures 3E, F**). The healthy control had more immature B cells, whereas the patients had more naive B cells. In addition to the central memory CD4^+^ T cells, four other types of T cells in the patient were more prevalent than those in the control. Compared with those of the healthy control, the PBMCs of the patient displayed decreased monocytes and increased NK cells.

**Figure 4:**
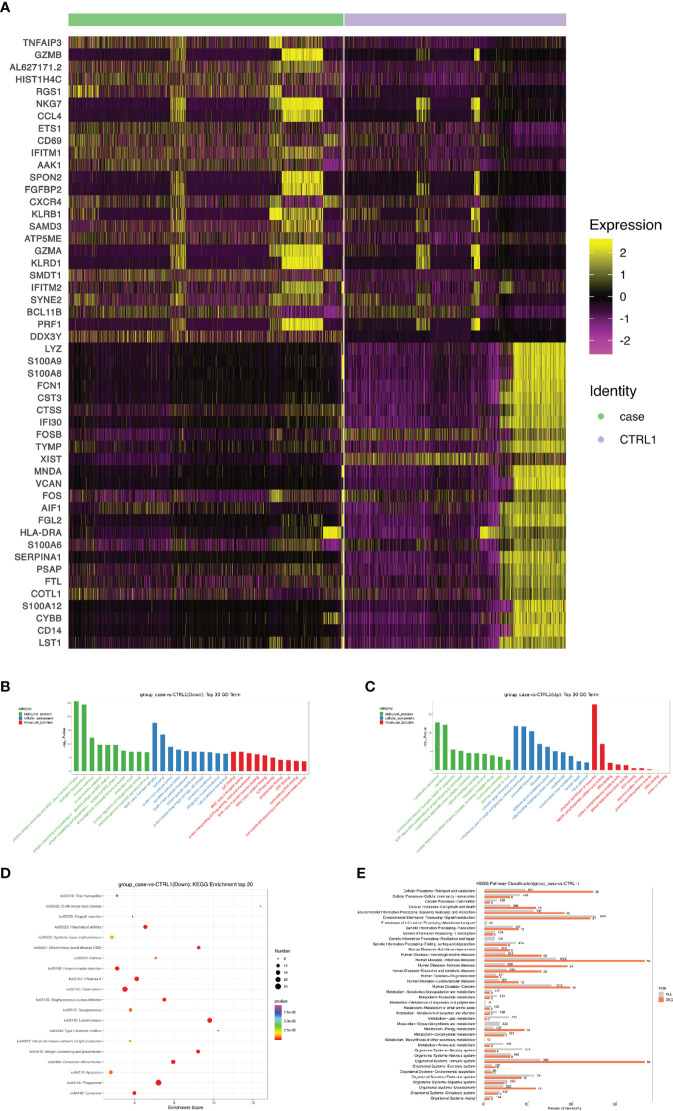
Screening and functional analysis of differentially expressed genes. **(A)** Heat map showing gene expression of the top 25 markers upstream or downstream (rows) detected in different cell populations from patient and healthy control (columns). **(B, C)** Selected Gene Ontology terms. Benjamini and Hochberg-corrected -log10 p values from hypergeometric tests. **(D)** The most enriched KEGG terms are ordered on the y-axis. X-axis represents the enrichment score in enriched KEGG terms. Sizes of the dots represent the number of genes included in each KEGG term. The color gradient of dots represents the adjusted P-values of each enriched KEGG term. **(E)** KEGG pathway enrichment analysis of DEGs. KEGG, Kyoto encyclopedia of genes and genomes; DEG, differentially expressed gene.

A heat map is used to illustrate highly expressed marker genes in each cluster (**Figure 3B**), and violin plots show the expression of several widely used marker genes in each cluster (**Figure 3C**). GO and KEGG enrichment analyses were performed to explore the biological processes of the differentially expressed genes (DEGs) in PBMCs between the patient and healthy control (**Figures 4B–E**). Pathway enrichment analysis revealed the downregulated DEGs with enrichment of terms such as peptide antigen assembly with MHC class II protein complex, neutrophil degranulation, immune response, and lysosome (**Figure 4B**), whereas upregulated DEGs were enriched in cytoplasmic translation, translation, cytosolic large ribosomal submit, cytoplasmic side of rough endoplasmic reticulum membrane, polysomal ribosome, and structural constituent of ribosome (**Figure 4C**). The roles of these genes require further investigation.

**Figure 5:**
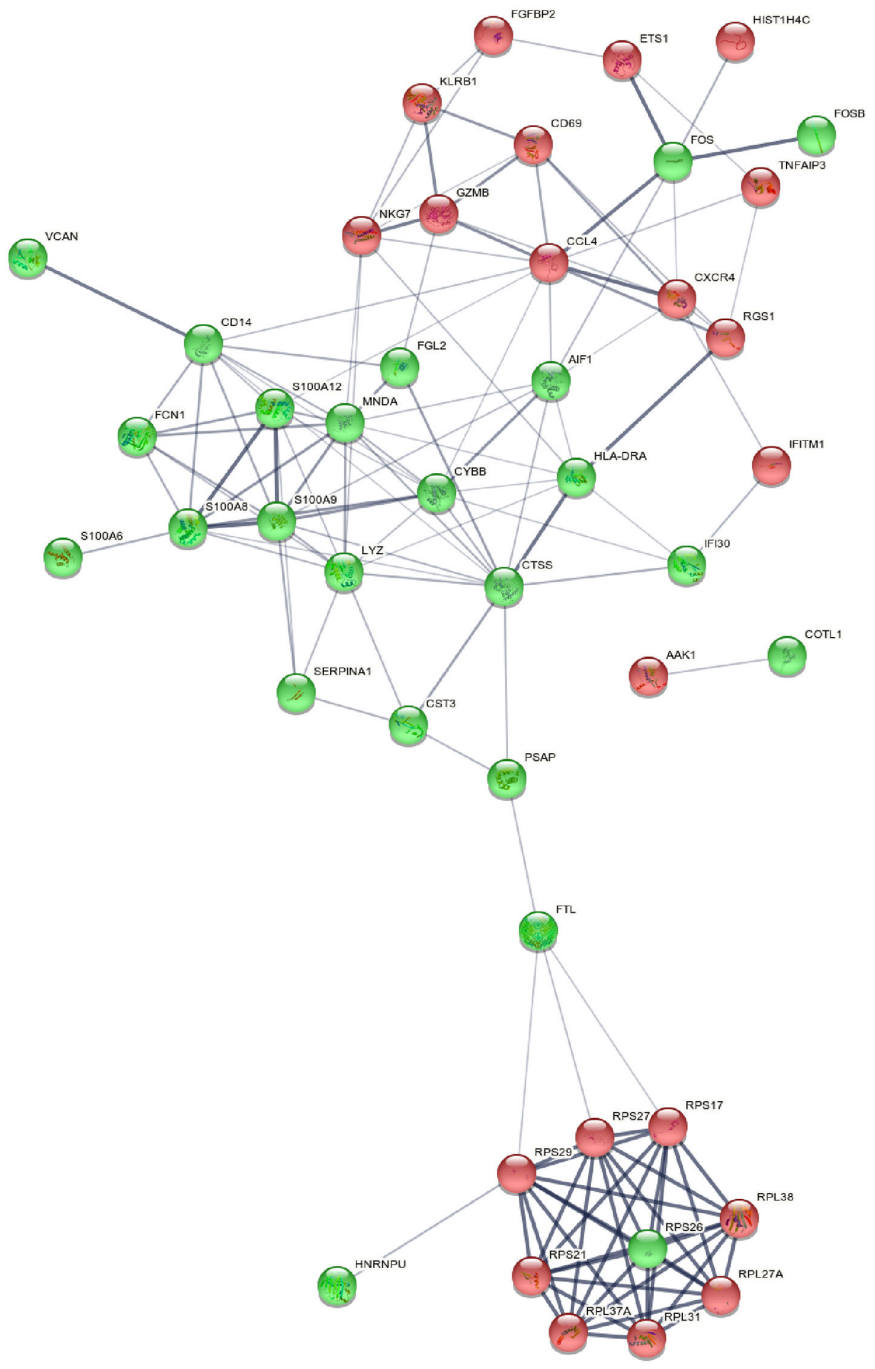
Picture of PPIN. PPIN was constructed by all the 45 DEGs using STRING database The nodes meant proteins; the edges meant the interaction of proteins; green circles meant down-regulated DEGs and red circles meant up-regulated DEGs. PPIN, protein-protein interaction network; DEG, differentially expressed gene; STRING, search tool for the retrieval of interacting genes.

Next, we sought to discover the immune molecular signatures associated with CMC by comparing DEGs between the CMC patient and healthy control (**Figures 4A** and **5**). Compared with that in the PBMCs of the control, the regulator of G protein signaling 1 (RGS1) in the PBMCs of the patient was significantly upregulated. RGS1 plays an important role in regulating the localization of lymphocytes and monocytes/macrophages, and can also regulate B cell homing to lymph nodes through chemokine signaling. *Rgs1^-/-^* mice spontaneously form germinal centers in the spleen, and the immune response was more robust and durable (18, 19). Inhibition of RGS1 in a mouse tumor model promoted the infiltration of effector T cells, further illustrating the effect of RGS1 on lymphocyte migration (20). There is a STAT1 binding site upstream of the transcription initiation site of RGS1, and activation of the INF–STAT1 pathway allows STAT1 to bind to the promoter of RGS1 to induce the transcription of RGS1. Researchers found that in CTL and Th1 cells high activity of STAT1 activates RGS1 transcription. Binding of RGS1 to G protein-coupled receptor accelerates the hydrolysis of GTP to GDP and then inactivates downstream signaling pathways, thereby inhibiting chemokine receptor function and resulting in reduced cell migration and survival (20, 21).

The expression of tumor necrosis factor alpha-induced protein 3 (TNFAIP3) in patient is significantly upregulated. TNFAIP3, also known as A20, is a ubiquitin-editing enzyme that suppresses inflammation by deubiquitinating TRAF6, and suppresses immune responses by blocking the NF-κB signaling pathway (22). A20 has the potential to suppress innate immunity, because A20^-/-^ bone marrow-derived macrophages exhibit excessive LPS-induced NF-κB and cytokine responses (23), and A20 can inhibit a subset of Toll-like receptor signaling and signaling triggered by the intracellular microbial sensor N2 (23, 24). Impaired A20 function may lead to autoimmune diseases. Previous studies have shown that A20 expression is lower in the PBMCs of patients with haploinsufficiency of A20 (HA20), which is typically manifested as Behçet’s-like disease. *In vitro* experiments in PBMCs of HA20 patients have shown that A20 not only inhibits inflammation through the NF-kB pathway, but also inhibits the STAT1 and IFN-γ pathways (25). In addition, A20 plays a negative regulatory role in the secretion of IL-17 by Th17 cells (26), so TNFAIP3 may be a potential pathogenic mechanism involved in CMC, although research is needed to investigate this.

S100A8 (MRP8) and S100A9 (MRP14), members of the S100 family of Ca^2+^-binding proteins, are constitutively expressed and secreted in neutrophils and monocytes, and are involved in innate immunity and the induction of inflammatory cytokines, reactive oxygen species, and nitric oxide to regulate inflammatory responses. As members of the antimicrobial peptides, S100A8 and S100A9 have antimicrobial properties and are chemotactic for neutrophils and monocyte-macrophages (27–29). Treatment with S100A8/A9 heterodimer increases the phagocytic capacity of human monocyte-derived macrophages and modulates phenotypic changes of macrophages (30). Moreover, type 17 immunity regulates S100A8 and S100A9 expression. IL-22 and IL-17A or IL-17F synergistically induce the expression of β-defensin 2 and S100A9, and enhance the expression of S100A8 additionally (31).

Compared with that in the healthy control, the expression of cathepsin S (CTSS) in our CMC patient was significantly downregulated. CTSS is mainly expressed in antigen-presenting cells such as B cells, macrophages, and dendritic cells, and it regulates antigen processing and presentation, as well as the T cell-mediated immunity response (32). The decreased expression of CTSS would affect the activity of proteases and related enzymatic immune responses. Exogenous antigens are taken up by antigen-presenting cells and degraded into short peptides suitable for MHC class II molecules. CTSS is mainly involved in the degradation of the invariant chain (Ii) in the MHC II-Ii complex, exposing the antigen peptide binding sites of MHC class II molecules and allowing for the binding of antigenic peptides, with the newly formed complexes being presented on the surface of CD4^+^ T cells (33–35).

### Review of the literature

We performed a literature review of articles published in English involving the STAT1 GOF mutation (c.Y289C). We used the following keywords to search electronic databases (Pubmed, Embase, Web of Science): “STAT1”, “Signal Transducer and Activator of Transcription 1”, “GOF”, and “gain of function”. We screened abstracts and full texts to identify all suitable articles. To be eligible for inclusion, case reports and studies had to describe the STAT1 GOF mutation (c.Y289C) carriers that had undergone genetic analysis. We reviewed articles cited in the literature to ensure the accuracy of the retrieved texts. Finally, two articles were included in the study (**Table 1**).

**Table 1:** Clinical characteristics of seven patients with STAT1 mutations (p.Y289C) located in CCD.

Authors/Year of publication	Age	Gender	Country	CMC	Age of first CMC	Other clinical symptoms
Toubiana et al 2016 (15)	20	F	France	+	Birth	Bacterial infections* Viral infections* Aneurysm
	14	F	France	+	11	Bacterial infections* Atopy
	36	M	UK	+	11	Bacterial infections* Viral infections
Vargas-Hernández et al 2018 (16)	5	M	ND	+	ND	CMV viremia HSV gingivostomatitis Recurrent thrush *Pneumocystis* jirovecii pneumonia
	8	M	ND	–	–	–
	30	F	ND	+	ND	–
This case	12	M	China	+	10	EBV infection Henoch-Schonlein purpura

*Probable or proven bacterial/viral infection; F, female; M, male; ND, not described; CMV, Cytomegalovirus; HSV, herpes simplex virus; EBV, Epstein-Barr virus. The symbols "+" means "exist" and "-" means “none”.

A total of seven patients (four males, three females) with the STAT1 GOF (c.Y289C) mutation were included in the selected studies (male/female ratio: 1.33). The median age of the seven patients at the time of the study was 14 years (range: 5–30 years), with an average age of 17.9 years. The median age at CMC onset (n=4, data lacking for three cases) was 10.5 years (range: birth to 11 years), while the median age at CMC onset in STAT1 GOF mutation carriers in the latest published systematic review (n=96, data lacking for 346 cases) was 1.0 (range: birth to 30 years) (36). Overall, the c.Y289C mutation appeared to be associated with later onset of CMC, which may explain why one of the 8-year-old male mutation carriers had not yet developed CMC. Viral and bacterial infections were common clinical manifestations in six patients who had developed CMC, with 66.7% (n=4) presenting viral infections and 50% (n=3) presenting bacterial infections, which was similar to a previous study (36). In addition, 33.3% (n=2) exhibited autoimmune predisposition (atopy, Henoch–Schonlein purpura), 16.7% (n=1) had an aneurysm, and 16.7% (n=1) had *Pneumocystis jirovecii* pneumonia.

## Discussion

In this study, we employed single-cell transcriptomic analysis to gain greater insight into the pathology of CMC than routine clinical tests, to explore potential genes of interest through the analysis of the DEGs of PBMCs, and to more comprehensively characterize the immune response in a CMC patient harboring a STAT1 GOF mutation. The single-cell analysis results generally reflected the patient’s immunodeficiency in antigen presentation and antibacterial effects, which was consistent with previous research.

Single-cell analysis revealed several DEGs related to immune regulation in this patient, including *RGS1*, *TNFAIP3*, *S100A8/A9*, and *CTSS*. By combining whole-exome sequencing and single-cell sequencing results, we observed marked upregulation of RGS1 transcript levels in our patient harboring the STAT1 GOF mutation. RGS1 is a member of the G protein signaling regulator (RGS) family. Previous studies have shown that activation of the INF–STAT1 pathway by some immune cells (such as CTL and Th1 cells) in the breast cancer tumor microenvironment can promote the upregulated transcription of RGS1. Chemokine receptors normally signal through intracellular G proteins, and RGS1 regulates T cell migration by attenuating chemokine-mediated signaling. However, the negative regulatory role of RGS1 stimulated by STAT1 during the immune response against fungal infection remains to be further studied.

CMC is clinically characterized by recurrent or persistent infections of the skin, nails, and mucous membranes with *Candida* (1). CMC usually presents with severe immunodeficiency of T cells, mainly associated with an impaired Th17 cell pathway. The STAT family regulates the transcription of multiple genes, leading to the increased production of IL-6, IL-10, IL-17A/17F, IL-22, transforming growth factor β, and monocyte chemoattractant protein 1, and the decreased production of tumor necrosis factor (TNF) α, IL-12, and IFN-γ (37). The STAT1 GOF mutation leads to the impaired dephosphorylation or hyperphosphorylation of STAT1 in the nucleus. Most of the mutation sites are located in the CCD and DBD of the nucleus, with a few being located in the SH2 domain. The reason why the STAT1 GOF mutation is not conducive to Th17 cell production and the IL-17 response, may be that it inhibits the downstream STAT3-mediated gene expression of Th17 cell growth and development, such as the STAT3 signaling pathway activated by IL-6, IL-21, and IL-23 (38), whereas, IFN-α, IFN-β, and IL-27 responses inhibit Th17 cell development (39, 40).

STAT1 GOF mutations not only cause infection but also cause other non-infectious symptoms, such as deep fungal diseases such as coccidioidomycosis and histoplasmosis, an increased susceptibility to bacterial sinus and lung infections, mycobacteria infection, and herpes virus infection (41–43). The STAT1 GOF mutation is also commonly associated with autoimmune diseases, such as autoimmune thyroid disease, autoimmune agranulocytosis, autoimmune hepatitis, vitiligo, type I diabetes, and systemic lupus erythematosus-like disease (13, 14).

In our review of the literature on STAT1 GOF (c.Y289C) mutations, we identified seven cases in total. With the exception of an 8-year-old boy who did not show CMC, approximately half of the remaining cases had bacterial and viral infections (such as CMV, HSV, and EBV). In previous STAT1 GOF studies, high rates of bacterial (74%) and viral (38%) infection were detected, with common viral infectious agents being HSV, varicella-zoster virus, CMV, and EBV (15, 36). Two patients showed autoimmune tendencies, one patient had an invasive infection, and one had an aneurysm. There were no reports linking tumor development to the c.Y289C mutation. Except for the older age of initial onset of CMC, the incidence of pre-existing clinical disease in c.Y289C mutants was similar to previous data (36). Overall, patients with STAT1 GOF (c.Y289C) mutations showed lower mortality and lower probability of failure to thrive (approximately 13.3% and 15.4% for STAT1 GOF, respectively) (36).

The molecular mechanism of STAT1 hyperphosphorylation was previously thought to be nuclear dephosphorylation injury (44). Because increased STAT1 protein levels and increased mRNA expression were observed in CMC patients, Bernasconi *et al.* suggested that the increased STAT1 phosphorylation levels resulted from impaired dephosphorylation on the one hand and increased total STAT1 protein levels on the other hand (45). Tamaura *et al.* demonstrated the high levels of STAT1 mRNA and protein expression in a mouse model knocked in with the R274Q mutation (46). Unlike some previous studies, however, we did not observe an increase in the protein level of STAT1 in our patient compared with the level in the healthy control.

Clinical diagnosis of CMC is usually based on clinical symptoms, and smear and *in vitro* culturing of *Candida*, but can also be based on gene sequencing analysis. In a large cohort study (n=273), CD8^+^ T cell counts were found to be normal in 83% of patients with STAT1 GOF mutations, decreased in 16%, and increased in only 1%. Memory B cells were reduced in 49% of the 53 patients tested, yet the results of the current study were insufficient to explain the rare elevation of CD8^+^ T cells in this patient. In fact, immunological laboratory findings are insufficient to diagnose the functional deficit in most patients. Although the patient’s monocyte count was within the normal range on laboratory tests, we noted a marked decrease in single-cell results compared with those of healthy controls. Studies suggest that STAT1 may be involved in the polarization of classical monocytes that drive the inflammatory response upon microbial infection. Recently, researchers observed an increase in phosphorylated STAT1 and total STAT1 levels in LPS-depleted monocytes, and further studies suggest that TRAM-mediated STAT1 activation leads to monocyte exhaustion (47)

At present, the first-line drugs for the treatment and prevention of CMC are azole antifungal drugs, among which fluconazole is the typical representative, followed by itraconazole and posaconazole. The recommended dose of fluconazole is 100–200 mg per day, and the dose can be increased to 800 mg per day for systemic infections and reduced to 6–12 mg/kg per day for children (48). In previous studies, the outcomes with fluconazole treatment were excellent in patients without other non-infectious diseases such as autoimmunity disease, aneurysm, or cancer. However, the susceptibility of *Candida* to fluconazole decreases with the duration of treatment, so it is necessary to isolate the strains regularly for drug susceptibility testing (49). Most patients with STAT1 GOF mutations require long-term local or systemic antifungal therapy, of which approximately 39% of patients with long-term antifungal therapy develop resistance to at least one antifungal agent, and patients require second- and third-line therapy, such as voriconazole, echinocandin, terbinafine, and liposomal amphotericin B (15).

Granulocyte-macrophage colony-stimulating factor (GM-CSF) and macrophage colony-stimulating factor (M-CSF) can promote Th17 differentiation and are second-line treatments for severe and invasive fungal infections in CMC (50). In 1995, Shahar *et al.* first reported that a CMC patient with a STAT1 GOF mutation obtained significant clinical benefit after receiving GM-CSF treatment (51).

In addition, some CMC patients can be treated with oral tyrosine kinase (JAK) inhibitors, such as the JAK1 and JAK2 inhibitor ruxolitinib. By targeting the JAK/STAT axis, JAK inhibitors are promising drug candidates in the treatment of STAT1 GOF or STAT 3 GOF mutations. Ruxolitinib inhibits the hyper-responsiveness of STAT1 to ligand stimulation, triggers Th1 and follicular T helper cell immune responses (52), and partially restores NK cell differentiation and function (16). A patient with a STAT1 GOF mutation showed significant improvement following administration of baricitinib and IL-17A production by PBMCs was partially restored (53).

*In vitro* studies on histone deacetylase inhibitors demonstrated their potential for clinical treatment of CMC. STAT1 acetylation counteracts IFN-induced STAT1 phosphorylation, nuclear translocation, DNA binding, and target gene expression (54).

At present, hematopoietic stem cell transplantation is the only method that can completely cure severe or refractory CMC, but there are associated risks, such as uncontrollable infection, graft-versus-host reactions, and death after secondary transplantation failure (17, 55). It is the responsibility of clinicians to judge whether patients are suitable for bone marrow transplantation and the optimal time for transplantation.

A limitation of this study is the small number of CMC cases, and future studies with a larger sample size are needed to reduce bias and reveal discrepancies between patients. Because CMC incorporates a wide spectrum of diseases and involves a range of pathogenic gene mutations (such as mutations in *STAT1*, *STAT3*, *IL-17F*, *IL-17RA/RC*, *ACT1*, and *RORC*), single-cell transcriptomic studies with more samples classified according to different mutated genes would be more meaningful, as well as sampling before and after GM-CSF treatment in patients. In addition, data based solely on bioinformatic technology need to be subsequently verified by cell or animal experiments.

In summary, we depicted the PBMC landscape in a CMC patient with a STAT1 GOF mutation. Our study established the feasibility of single-cell RNA-Seq technology as a strategy to investigate immune pathogenic responses in CMC.

## Data availability statement

The raw sequence data have been deposited in the Genome Sequence Archive (Genomics, Proteomics & Bioinformatics 2021) in National Genomics Data Center (Nucleic Acids Res 2021), China National Center for Bioinformation / Beijing Institute of Genomics, Chinese Academy of Sciences (GSA: HRA002902), accessible at https://ngdc.cncb.ac.cn.

## Ethics statement

The studies involving human participants were reviewed and approved by Committee on Ethics of Medicine, Naval Medical University, PLA. Written informed consent to participate in this study was provided by the participants’ legal guardian/next of kin. Written informed consent was obtained from the individual(s), and minor(s)’ legal guardian/next of kin, for the publication of any potentially identifiable images or data included in this article.

## Author contributions

LD, XGL, and WP contributed to conception and design of the study. XDL, KZ, and WJ wrote the first draft of the manuscript. HL, YH, MD, JW, and YC wrote sections of the manuscript. All authors contributed to manuscript revision, read, and approved the submitted version.

## Funding

This work was supported by the National Natural Science Foundation of China (82072257), Chinese Academy of Engineering (2022-HZ-10-3) and Project of Clinical Outstanding Discipline Construction in Shanghai Pudong New Area (PWYgy2021-09). We thank OE Biotech Co., Ltd (Shanghai, China) to help us in sequencing analysis.

## Conflict of interest

The authors declare that the research was conducted in the absence of any commercial or financial relationships that could be construed as a potential conflict of interest.

## Publisher’s note

All claims expressed in this article are solely those of the authors and do not necessarily represent those of their affiliated organizations, or those of the publisher, the editors and the reviewers. Any product that may be evaluated in this article, or claim that may be made by its manufacturer, is not guaranteed or endorsed by the publisher.
